# BRCA2 and TP53 Mutations in a Breast Cancer Patient: A Case Report and Review of the Literature

**DOI:** 10.7759/cureus.71310

**Published:** 2024-10-12

**Authors:** Jackeline Lopes Viana, Elisa Rosa de Carvalho Gonçalves Nunes, Cristiane Amaral dos Reis, Sabas Carlos Vieira

**Affiliations:** 1 Mastology, Oncocenter, Teresina, BRA; 2 Clinical Oncology, Oncocenter, Teresina, BRA; 3 Tocogynecology, Oncocenter, Teresina, BRA

**Keywords:** brca gene, breast cancer, hereditary cancer syndrome, li fraumeni syndrome, tp53 gene

## Abstract

Hereditary breast cancer (BC) accounts for 5-10% of all BC cases. Pathogenic or likely pathogenic germline variants in *BRCA1* and *BRCA2* genes are the most common cause of hereditary BC. However, other genes may also be involved, such as *TP53*, *PALB2*, *PTEN*, *ATM*, and *CHEK2,* among others. Multigene tests are essential in the treatment approach to young BC patients, since the detection of specific mutations may help guide changes in preventive measures and treatment plans. This report describes a rare case of BC in a young patient with pathogenic germline variants in *BRCA2* and *TP53 *genes and also presents a literature review of the topic.

## Introduction

Breast cancer (BC) is the most commonly diagnosed cancer globally and it is the main cause of death from cancer among women worldwide [1]. Around 5-10% of BC cases are hereditary. Pathogenic and likely pathogenic germline variants in* BRCA1/2* genes that predispose to hereditary breast, ovarian, and pancreatic cancer syndrome (HBOC) are the most frequent genetic conditions associated with this condition. Women carrying these genes have up to an 87% lifetime risk of developing breast and/or ovarian cancer [2]. Nevertheless, other genes are also related to hereditary BC and may be classified as highly or moderately penetrating genes with a lifetime risk of >30% or 17-30%, respectively. Examples of highly penetrating genes include *TP53*, *PTEN*, *CDH1*, *STK11,* and *PALB2*. Moderately penetrating genes include *CHEK2*, *ATM*, *BARD1*, *BRIP1*, *NBN*, *NF1*, *RAD51D,* and *MSH6* [3].

*TP53* is a tumor suppressor gene with a key role in cellular response to DNA damage. It induces pathways involved in apoptosis, cell cycle arrest, and DNA repair mechanism to maintain the genomic integrity of the cell, and is considered the guardian of the human genome. Germline mutations in *TP53* are rare and may meet the diagnostic criteria for Li-Fraumeni syndrome, which is characterized by an increasingly significant risk of female BC, bone or soft tissue cancers, brain tumors, and adrenocortical carcinomas. A recent study suggests that screening for *TP53* germline mutation helps detect a clinically significant risk of early-onset BC and should be considered in all young BC patients, even when they test negative for *BRCA1/2* gene mutations [4].

The concomitant occurrence of pathogenic germline variants in *TP53,* *BRCA1,* and *BRCA2* genes is rare [5]. We report a case of BC in a young patient with germline pathogenic variants (PVs) in *BRCA2* and *TP53* genes and engage in a review of the relevant literature.

The study is part of a research project approved by the State University of Piauí (CAAE 30154720.0.000.5209) and the patient signed a written informed consent term for the publication of the case.

## Case presentation

**Figure 1:**
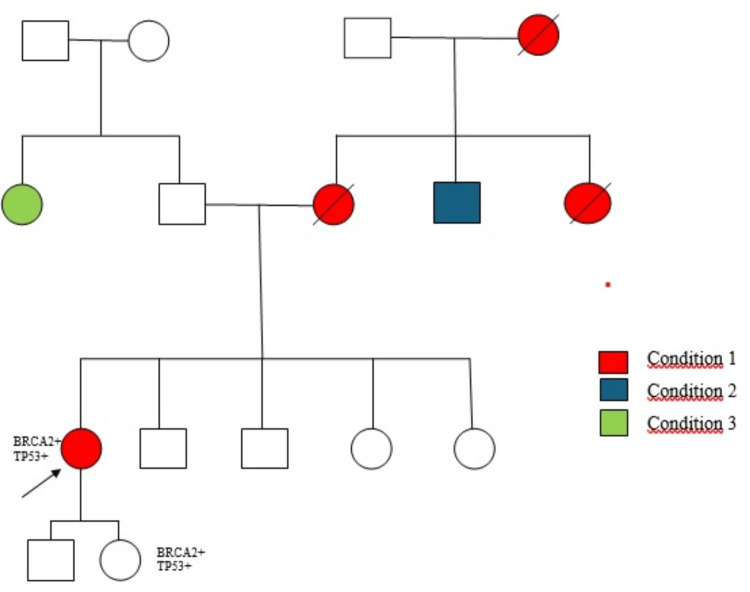
Familial pedrigree of the case The proband is indicated by an arrow. Squares represent males, and circles represent females. Slash symbols indicate deceased individuals. Color symbols represent affected individuals. Condition 1: breast cancer; condition 2: skin cancer; condition 3: unspecified cancer. The proband and her daughter carry mutations in *BRCA2* and *TP53* genes

The patient was a 40-year-old female who had been diagnosed with breast carcinoma at the age of 32 years when she already had two children. She had first given birth at the age of 28 years and had stopped breastfeeding her second child four months before her cancer diagnosis. Her menarche had been at the age of 15 years. There was no history of comorbid conditions; she had a sedentary lifestyle and she did not smoke or drink alcohol. Her family history included several cases of cancer: her mother had died at the age of 28 years from breast cancer; her maternal aunt and maternal grandmother had also died from breast cancer; her maternal uncle had skin cancer; and a paternal aunt had cancer of unknown origin (Figure 1).

At the initial consultation in September 2015, the patient reported a painless lump in her left breast two weeks previously. On physical exam, there was a palpable mass in her left breast's upper lateral quadrant (ULQ) measuring around 2 cm. There was no axillary lymph node enlargement that raised suspicion of malignancy. An ultrasound of the breast revealed a mass in the ULQ of the left breast, measuring 2.1 x 1.6 cm, with internal calcifications and irregular contours, which is classified as BIRADS 4. Histopathology study of the core needle biopsy showed grade 1 invasive carcinoma of no special type. Mammography revealed only dense breasts. Chest radiography, complete blood count, and other biochemistry parameters were normal. Ultrasound of the abdomen detected a hyperchoic solid liver mass, measuring 1 cm, compatible with hemangioma.

The patient underwent segmental resection of the left breast with a negative frozen section of the free surgical margins and sentinel lymph node resection. Histopathology study of the specimen showed grade 2 invasive carcinoma of no special type, measuring 1.5 x 0.8 cm, with extensive high-grade in situ component and lobular extension, free margins, without angiolymphatic invasion, and mild lymphocyte infiltration. The sentinel lymph node was positive for metastases (pT1pN1M0 - IIA). Immunohistochemistry study revealed that the tumor was estrogen receptor-positive (70%), progesterone receptor-positive (40%), and human epidermal growth factor type 2 receptor (HER2)-positive [score: (3+)]. The silver stain hybridization in situ (SISH) confirmed overexpression of HER2, classified as Hybrid luminal B (prognostic stage IB). The patient then underwent adjuvant chemotherapy with doxorubicin and cyclophosphamide (four cycles, every three weeks), followed by 12 weekly cycles of taxol, 14 cycles of trastuzumab, every three weeks, and breast and axillary radiation therapy. She continues to use tamoxifen 20mg/day. Ovarian suppression with LHRH analogue has been proposed but the Brazilian public health system does not provide this medication for the treatment of premenopausal breast cancer.

**Figure 2:**
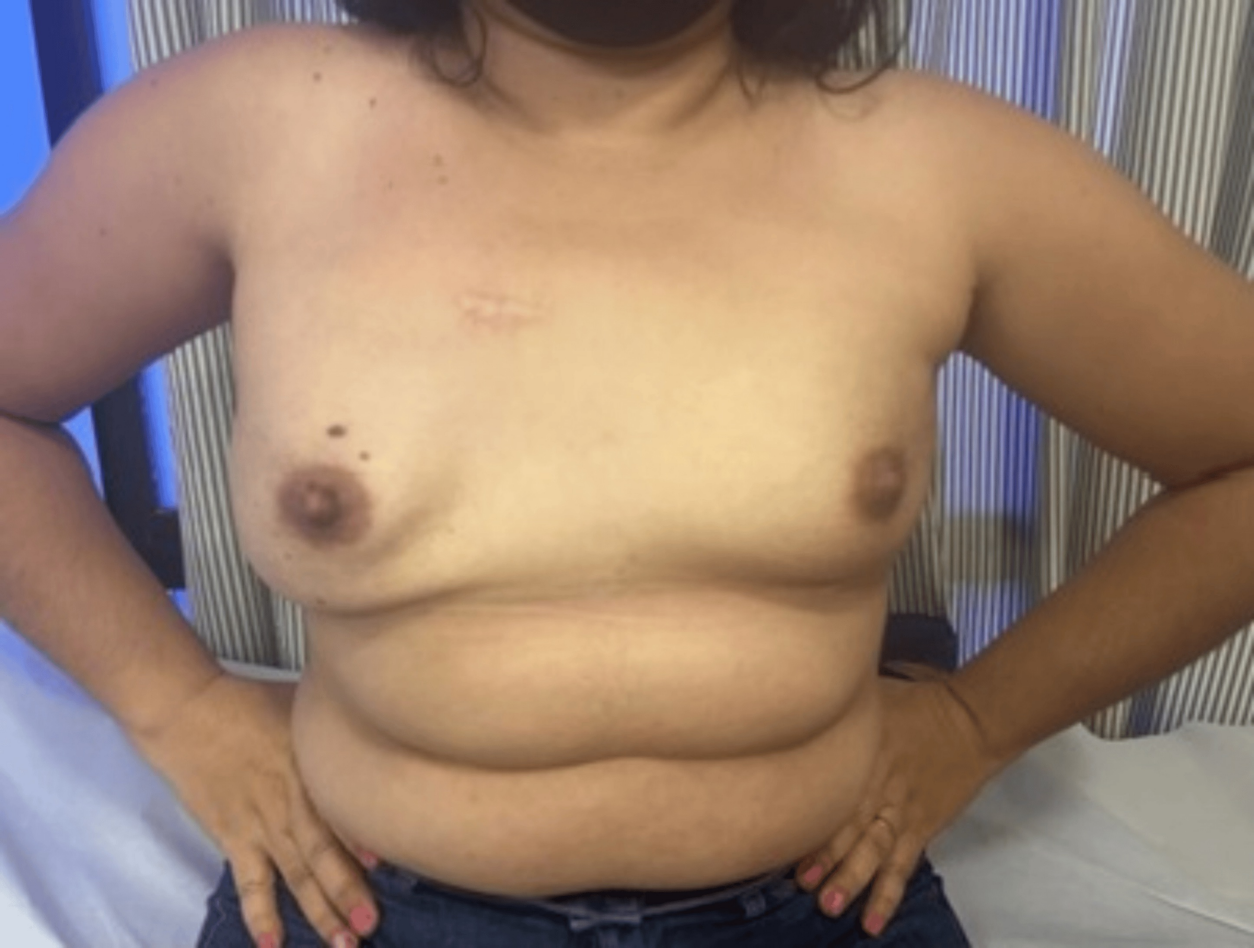
Postoperative image The patient underwent a two-step mastectomy (a reductive mammoplasty and then a risk-reducing mastectomy with the inclusion of a submuscular prosthesis)

Although the patient was advised at the first consultation to do a genetic test, she only underwent multigene panel testing four years after the diagnosis due to financial constraints. The multigene test for genes of hereditary predisposition to cancer detected *BRCA2* heterozygous pathogenic mutation (c.3116+5G>A(intronic)) and *TP53* (c.1010G>A(p.Arg337His)). In the following year, she underwent a two-step risk-reducing bilateral mastectomy (Figure 2). The surgical specimen was negative for malignancy in the histopathology study. The patient is currently undergoing preparation for bilateral salpingo-oophorectomy by videolaparoscopy. During eight years of follow-up, the patient has progressed without active disease. Her 10-year-old daughter also underwent a multigene test and is a carrier of the same pathogenic mutations as the mother in *BRCA2* and *TP53*. The patient was advised to undergo a whole-body MRI due to *TP53* gene mutation.

## Discussion

In the PubMed database, we found only one study that reported the association between *BRCA2* and *TP53* gene mutation (based on a search with the keywords breast cancer,* BRCA* gene, *TP53* gene, hereditary cancer syndrome, and Li Fraumeni syndrome). It is a Korean study involving 55 patients with pathogenic mutations in *BRCA1*/*2* genes (27 and 28 cases, respectively), undergoing genetic sequencing to investigate gene alterations related to homologous recombination. The most frequently found commutated gene was *TP53* (21/55, 38.1%), while *BRCA2* and *TP53* gene commutation occurred in nine cases. In that study, patients with* BRCA1*/*2* and *TP53* gene mutations had higher chances of developing triple-negative BC (p=0.028), and high Ki-67 values (p = 0.001). On the other hand,* BRCA2* and *TP53* gene commutation was associated with higher overall survival than those cases without the *TP53* gene mutation[6].

BRCA1/2 proteins are tumor suppressors that contribute to genomic stability through the repair of double-strand break mediated by homologous recombination, in cooperation with other proteins also related to homologous recombination. Germline mutations of these genes are associated with an increased lifetime risk of cancer in various organs, including breast, ovaries, colon, prostate, and pancreas [7]. On the other hand, Li Fraumeni syndrome is a rare autosomal dominant hereditary syndrome, characterized by germline mutations in the *TP53* gene that increase the risk of BC, mainly in younger women, and at 60 years, the cumulative incidence of BC may reach 85%. In Li Fraumeni syndrome, the majority of breast tumors are positive for estrogen receptors with frequent co-expression of HER2, as in the present case, which leads to the assumption that the BC may have been related to *TP53* gene mutation [8].

In a Brazilian cohort, the prevalence of Li-Fraumeni syndrome in BC patients was 3.5% (8/224) and represented 17.4% (8/46) of carriers of PVs; six of these patients (2.7%, 6/24) were carriers of the Brazilian variant-*TP53 *(R337H). This same study observed that the detection of PVs was statistically significantly associated (p<0.05) with a diagnosis of BC before the age of 45 years, high-grade tumors, bilateral BC, history of multiple primary cancers, and family history of pancreatic cancer, as in the present case [9]. In the south and southeast of Brazil, the prevalence of R337H mutation is one for every 300 people born [10]. In the present case, although the patient was born in Piaui, her mother was from Paraná, a state in southern Brazil. Genetic mutation originating from that region was carried by the “route of the troops” and disseminated to other states[11].

A systematic review has shown a significant molecular heterogeneity among mutations in patients with HBOC in Brazil. Three mutations are highlighted: c.5266dupC, c.156_157insAlu, and c.1010G>A in* BRCA1*,* BRCA2, *and *TP53* genes, respectively. Together, these three mutations appear in more than 200 registrations and seem to have a vital role in the pathology of breast and ovarian cancer in Brazil. However, the association between *BRCA2* and *TP53 *mutation has not been reported [12]. As per the National Comprehensive Cancer Network (NCCN), the follow-up of women with pathogenic mutation of the *BRCA2* gene involves a monthly self-exam of the breasts beginning at 18 years and a physical exam of the breast every 6-12 months beginning at 25 years; from age 25 to 29 years, screening should be performed with MRI of the breasts with or without contrast (or mammography, only if MRI is unavailable), since MRI has a high sensitivity to tumor detection and decreases exposure to radiation in this age group. From 30 to 75 years, mammography and MRI should be performed annually; in those aged >75 years, follow-up is individualized. When mammography is performed, it should be associated with breast tomosynthesis to increase sensitivity and lower false-positive rates. These same guidelines should be followed in women who had BC and did not undergo bilateral mastectomy.

Counseling after a positive test for *BRCA2* should involve explaining the risk of developing BC at an earlier age and the increased risk of developing contralateral BC. The patient should also be advised about risk reduction, conferred by risk-reducing bilateral mastectomy and/or bilateral risk-reducing salpingo-oophorectomy (between ages 40 and 45 years), although each case should be individualized, taking into account age, life expectancy, and family history. Chemoprevention with selective estrogen receptor modulators and aromatase inhibitors, although proven to be efficient at reducing the risk in patients classified as high-risk for BC, requires further studies in patients with pathogenic and likely pathogenic variants in *BRCA1*/*2* genes [13].

Since *TP53* germline mutations are related to an increased risk of early-onset BC, screening and follow-up recommendations are the same for patients with *BRCA2* mutation. Nevertheless, screening should begin earlier, at the age of 20 years, or at the age at which the youngest relative was diagnosed with BC. Similarly, MRI is preferable in a younger age group (20-29 years), and when mammography is performed (after 30 years of age), it should be followed by tomosynthesis. Risk-reducing mastectomy should also be considered and discussed individually. Furthermore, there is no evidence for chemoprevention in this group of patients [13]. Patients with *TP53* gene mutation should receive radiotherapy, except when an alternative does not exist. In patients with breast carcinoma, the recommended treatment is mastectomy with sentinel node investigation, to avoid radiotherapy in early-onset cases such as in the present case. Radiotherapy is associated with an increased risk of radiation-induced cancers [14,15]. Our patient did not undergo genetic testing at the time of adjuvant radiotherapy.

In addition, in the treatment of BC patients with *BRCA1*/*2* mutations, it is possible to include the use of inhibitors of poly(adenosine diphosphate-ribose) polymerase (PARP) enzyme, as *BRCA1/2* are tumor suppressor genes involved in transcription regulation and repair of double-strand breaks (DSB) in the DNA molecule, playing a key role in the homologous recombination pathway. Cells with loss of function in these genes are unable to repair DNA errors and start to rely on PARP capacity to detect damage and activate alternative repair pathways, thereby maintaining genome integrity. Functional loss of either gene BRCA or PARP may be well-tolerated, but inhibition of PARP function in cells with* BRCA* gene mutations makes them incapable of repairing DNA damage, leading to cancer cell death (synthetic lethality) [16,17]. At the time of adjuvant therapy, the patient had not received the result of the genetic test, and olaparib is not available in the Brazilian public health system.

Therefore, in patients who meet the criteria of suspicion for hereditary breast cancer, multigene tests can help detect mutation in more than one gene associated with different hereditary syndromes. With the help of these findings, it is possible to establish proper management for risk reduction and specific treatment, including the use of PARP inhibitors in carriers of *BRCA2* gene mutation and prevention of radiation therapy in patients with a pathogenic mutation of the *TP53 *gene [18-20]. The present report highlights the importance of multigene tests for young BC patients.

## Conclusions

Hereditary BC should be considered in cases of BC in young women with a significant family history of malignant neoplasms. Multigene tests should be offered to these patients, as there may be mutations in more than one gene, as in this case report of a young patient with a rare comutation in *BRCA2* and *TP53* genes. Early diagnosis of genetic mutations is key to optimizing the treatment and follow-up of patients with hereditary BC.
